# Metabolic engineering of *Saccharomyces cerevisiae* for high-level production of gastrodin from glucose

**DOI:** 10.1186/s12934-020-01476-0

**Published:** 2020-11-26

**Authors:** Hua Yin, Tiandong Hu, Yibin Zhuang, Tao Liu

**Affiliations:** 1 Tianjin Institute of Industrial Biotechnology, Chinese Academy of Sciences, Tianjin, 300308 China; 2 Key Laboratory of Systems Microbial Biotechnology, Chinese Academy of Sciences, Tianjin, 300308 China

**Keywords:** *Saccharomyces cerevisiae*, Gastrodin, AsUGT, Phenolic glycoside, Chromosomal integration, Metabolic engineering

## Abstract

**Background:**

The natural phenolic glycoside gastrodin is the major bioactive ingredient in the well-known Chinese herb Tianma and is widely used as a neuroprotective medicine in the clinic. Microbial production from sustainable resources is a promising method to replace plant extraction and chemical synthesis which were currently used in industrial gastrodin production. *Saccharomyces cerevisiae* is considered as an attractive host to produce natural plant products used in the food and pharmaceutical fields. In this work, we intended to explore the potential of *S. cerevisiae* as the host for high-level production of gastrodin from glucose.

**Results:**

Here, we first identified the plant-derived glucosyltransferase AsUGT to convert 4-hydroxybenzyl alcohol to gastrodin with high catalytic efficiency in yeast. Then, we engineered de novo production of gastrodin by overexpressing codon-optimized AsUGT^*syn*^, the carboxylic acid reductase gene *CAR*^*syn*^ from *Nocardia* species, the phosphopantetheinyl transferase gene *PPTcg-1*^*syn*^ from *Corynebacterium glutamicum*, the chorismate pyruvate-lyase gene *UbiC*^*syn*^ from *Escherichia coli*, and the mutant *ARO4*^*K229L*^. Finally, we achieved an improved product titer by a chromosomal multiple-copy integration strategy and enhancement of metabolic flux toward the aglycon 4-hydroxybenzyl alcohol. The best optimized strain produced 2.1 g/L gastrodin in mineral medium with glucose as the sole carbon source by flask fermentation, which was 175 times higher than that of the original gastrodin-producing strain.

**Conclusions:**

The de novo high-level production of gastrodin was first achieved. Instead of chemical synthesis or plants extraction, our work provides an alternative strategy for the industrial production of gastrodin by microbial fermentation from a sustainable resource.

## Background

The phenolic glycoside gastrodin is the major bioactive ingredient in the famous Chinese herb Tianma (the tuber of *Gastrodia elata* Blume), which has been widely used to treat nervous system diseases, such as ischemic stroke, epilepsy, dizziness, and dementia, for centuries in China [1, 2]*.* In addition to its neuroprotective effects [3], gastrodin has been proven to show memory improvement [4], anti-inflammation, and antioxidant effects [5, 6]. To date, gastrodin is produced mainly by chemical synthesis or direct extraction from Tianma, although different processing methods have also been explored and applied [7, 8]. However, total chemical synthesis is a complicated process and leads to severe environmental pollution due to the use of toxic phenols, phosphate and bromide. Direct extraction from Tianma is costly and time consuming because of the low gastrodin content in Tianma (below 0.7%) [8]. Meanwhile, wild resources of Tianma are scarce, and its artificial cultivation is relatively complicated due to the nature of its symbiotic relationship with two compatible mycorrhizal fungi during seed germination and vegetative growth [9]. The success of heterologous production of plant-derived natural products in microorganisms provides an alternative strategy for producing gastrodin in an economical and environmentally friendly manner [10].

The native biosynthetic pathway of gastrodin in plant has still not been documented. In our previous work [11], we first achieved microbial biosynthesis of gastrodin by constructing an artificial pathway in *Escherichia coli*, beginning with the conversion of chorismate to 4-hydroxybenzoic acid (4-HBA) by chorismate pyruvate-lyase (UbiC). Then, 4-HBA was transformed to 4-hydroxybenzyl aldehyde by carboxylic acid reductase (CAR), which is activated into its holo form by a phosphopantetheinyl transferase (sfp). The 4-hydroxybenzyl aldehyde was transformed to 4-hydroxybenzl alcohol by endogenous alcoho9l dehydrogenases (ADHs). Finally, 4-hydroxybenzl alcohol was converted to gastrodin by the glycosyltransferase UGT73B6 from *Rhodiola*. With further rational engineering of the glycosyltransferase, the resulting *E. coli* strain produces approximately 0.5 g/L gastrodin. Our previous results strongly suggest that the activity of UGTs is critical for the improvement of gastrodin biosynthesis. Unfortunately, the native glucosyltransferase for gastrodin biosynthesis is unknown. Tsai et al*.* [9] reported that a unigene encoding glycosyltransferase might participate in the gastrodin native biosynthetic pathway. However, its sequence has not yet been published. To date, UGT73B6 from *Rhodiola* is the only glycosyltransferase that has been characterized to be functional in gastrodin synthesis.

As common microbial platforms, *E. coli* and *Saccharomyces cerevisiae* have been engineered to produce aromatic amino acid-derived (AAA-derived), chorismate-derived or shikimate-derived products [12]. As in *E. coli*, some successful strategies have also been used in yeast to enhance the production of these products to gram levels per liter [13, 14]. Liu et al*.* [15] constructed an *S. cerevisiae* platform strain with an AAA-derived *p*-coumaric acid yield of more than 10 g/L by rewiring carbon metabolism, achieving appropriate levels for large-scale production. Food-grade *S. cerevisiae* is considered an attractive host due to its superior capability of expressing enzymes requiring posttranslational modification, safety, robust fermentation and resistance to phage infection [16].

In this work, we explored the potential of *S. cerevisiae* as the host to produce gastrodin. We first identified a glucosyltransferase with high catalytic efficiency for gastrodin synthesis and constructed a gastrodin-producing yeast strain by pathway engineering. By a chromosomal integration strategy and metabolic flux enhancement toward the 4-hydroxybenzyl alcohol, we achieved high-level production of gastrodin. Engineered yeast strains could serve as the basis for creating an alternative method to produce gastrodin, instead of extraction from plant sources or chemical synthesis in the future.

## Results and discussion

### The glucosyltransferase AsUGT catalyzed gastrodin biosynthesis with high efficiency in yeast

**Fig. 1:**
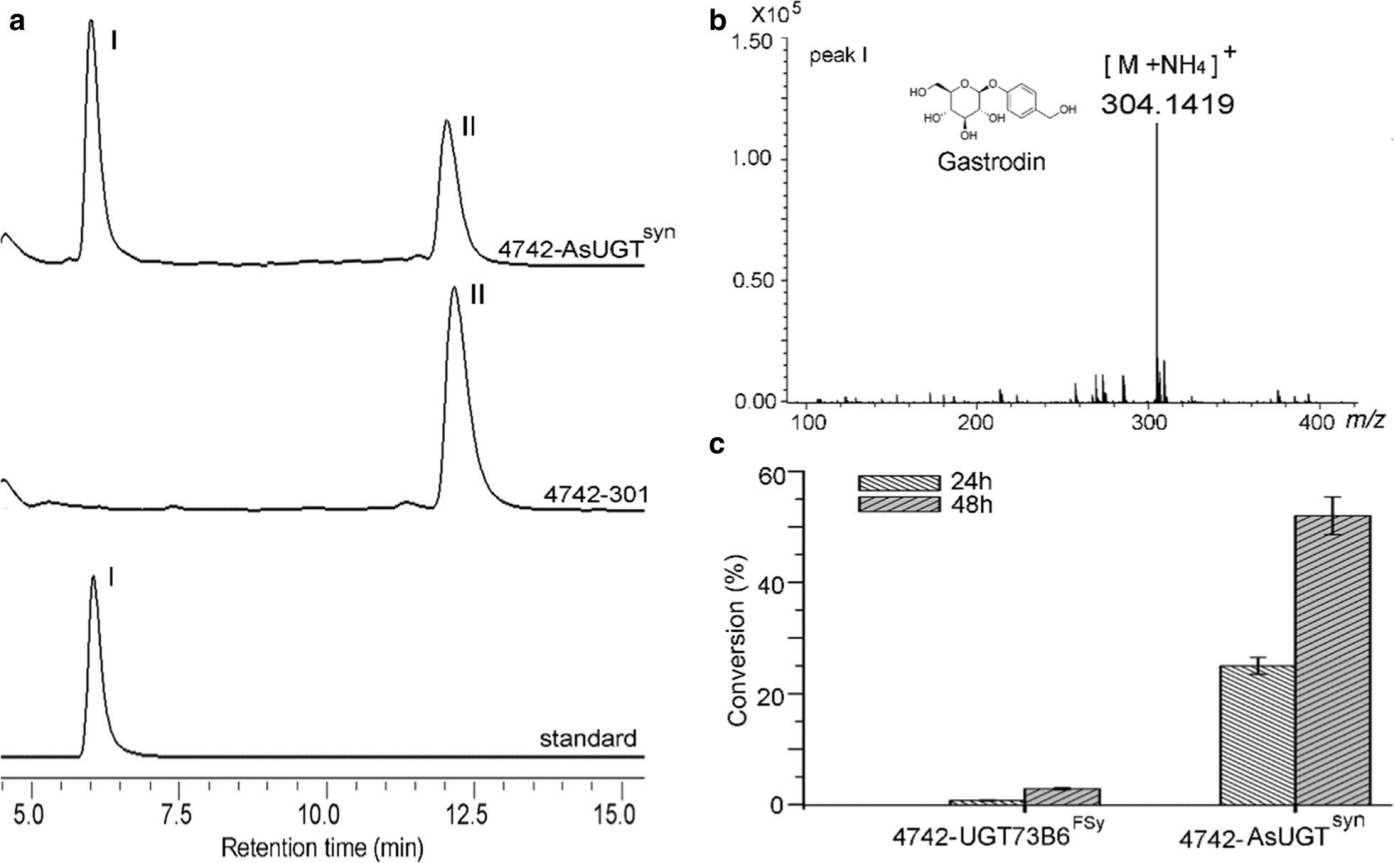
Identification of the AsUGT that catalyzes 4-hydroxybenzyl alcohol to gastrodin. **a** HPLC analysis of strain 4742-AsUGT^syn^ (carrying P_ScTEF1_-*AsUGT*^*syn*^ -T_CYC1_), control 4742–301 fermentation broth supernatants and standard gastrodin (I). Peak II was a 4-hydroxybenzyl alcohol substrate. **b** Peak I mass spectrum of the fermentation supernatant of strain 4742-AsUGT^syn^. **c** Bio-conversion rates of 4-hydroxybenzyl alcohol mediated by strain 4742-AsUGT^syn^ and 4742-UGT73B6^FSy^ (carrying P_ScTEF1_-*UGT73B6*^*FSy*^-T_CYC1_). Three replicates were performed, and the error bars represented standard deviation

To achieve heterologous biosynthesis of gastrodin in yeast, it is necessary to identify a compatible glucosyltransferase that recognizes 4-hydroxybenzyl alcohol as a substrate with high efficiency. It was reported that the glucosyltransferase AS from *R. serpentina* could accept phenols as substrates [17]. Based on the structural similarity between phenols and 4-hydroxybenzyl alcohol, we hypothesized that AS might be able to catalyze the glucosylation of 4-hydroxybenzyl alcohol. To test this hypothesis, we overexpressed a codon-optimized AS (named *AsUGT*^*syn*^ in this work) in yeast and carried out feeding experiments with 4-hydroxybenzyl alcohol as the substrate. HPLC analyses of the metabolites in the fermentation broth supernatants produced by the engineered strain displayed a new peak at a retention time of 6.3 min (Fig. 1a). HPLC–MS analysis showed that the new compound had a molecular ion at *m/z* 304.1419 ([M + NH_4_]^+^), which was identical to that of gastrodin (Fig. 1b). Approximately 0.5 mM gastrodin was produced within 24 h, and the substrate conversion rate was 25%. Within 48 h, the substrate conversion rate reached up to 55%, with approximately 1.1 mM gastrodin produced.

Our previous study demonstrated that the glucosyltransferase UGT73B6^FS^, a mutant of UGT73B6, showed high catalytic efficiency in the conversion of 4-hydroxybenzyl alcohol to gastrodin in *E. coli* [11]. To compare enzymatic efficiency between codon-optimized *AsUGT*^*syn*^ and *UGT73B6*^*FSy*^ in yeast, ScTEF1 promoter-driven *UGT73B6*^*FSy*^ was also introduced into *S. cerevisiae* BY4742. The resulting strain 4742-UGT73B6^FSy^ was cultivated under the same conditions. As shown in Fig. 1c, strain 4742-UGT73B6^FSy^ produced only trace amounts of gastrodin. The substrate conversion rate in strain 4742-AsUGT^syn^ was 33 times higher than that in strain 4742- UGT73B6^FSy^ within 24 h. Taken together, these results clearly showed that AsUGT exhibited a higher catalytic efficiency than UGT73B6^FS^ in yeast. Thus, AsUGT was used for heterologous gastrodin biosynthesis in subsequent experiments.

### De novo biosynthesis of gastrodin from glucose was achieved in *S. cerevisiae*

**Fig. 2:**
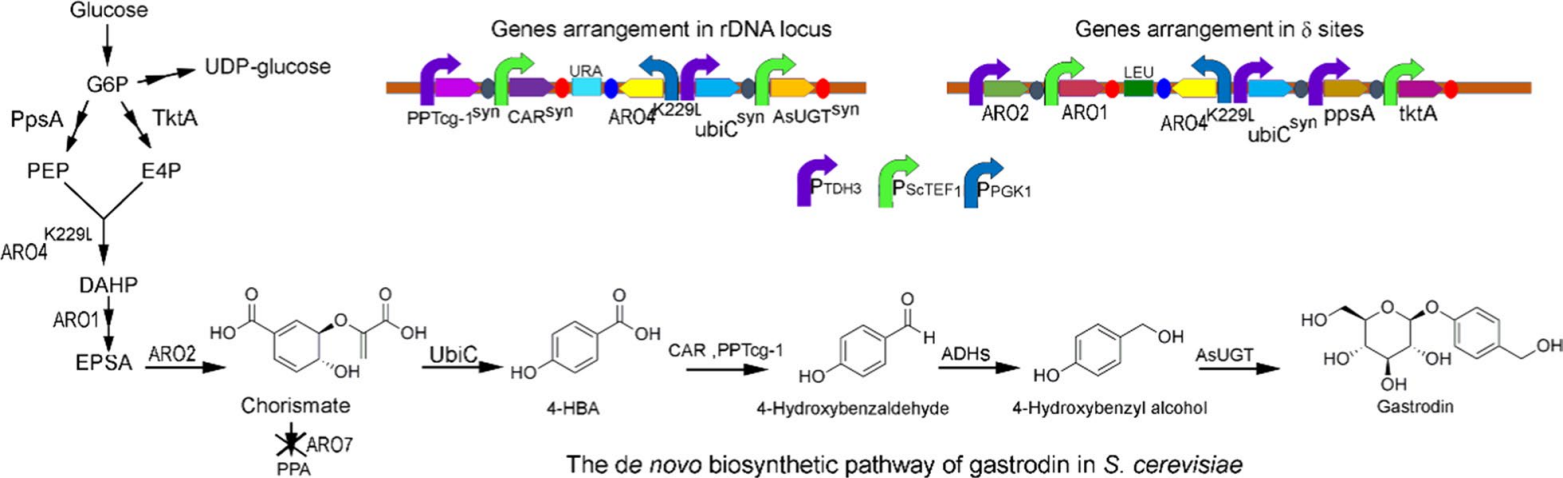
The *de no* biosynthetic pathway of gastrodin and genes arrangement in rDNA locus or *δ* sites. Single arrows represent one-step conversion, double arrows represent multi-step conversion, single arrow with cross lines represents pathway block. Abbreviations: PEP, phosphoenolpyruvate; E4P, erythrose 4-phosphate; DAHP, 3-deoxy-arabino-heptulonate-7-phosphate; EPSA, 5-enolpyruvyl-shikimate-3-phosphate; PPA, prephenate; 4-HBA, 4-hydroxybenzoic acid; PpsA, phosphoenolpyruvate synthase; TktA, transketolase; ARO4^K229L^, feedback-resistant DAHP synthase mutant; ARO1, pentafunctional EPSP synthase; ARO2, chorismate synthase gene; UbiC, chorismate pyruvate-lyase; CAR, carboxylic acid reductase; PPTcg-1, phosphopantetheinyl transferase; ADHS, alcohol dehydrogenases; AsUGT, glucosyltransferase; ARO7, chorismate mutase. P_TDH3_, TDH3 promoter; P_ScTEF1_, ScTEF1 promoter; P_PGK1_, PGK1 promoter. Genes with ‘^syn^’ represent codon-optimized genes in *S. cerevisiae*

**Fig. 3:**
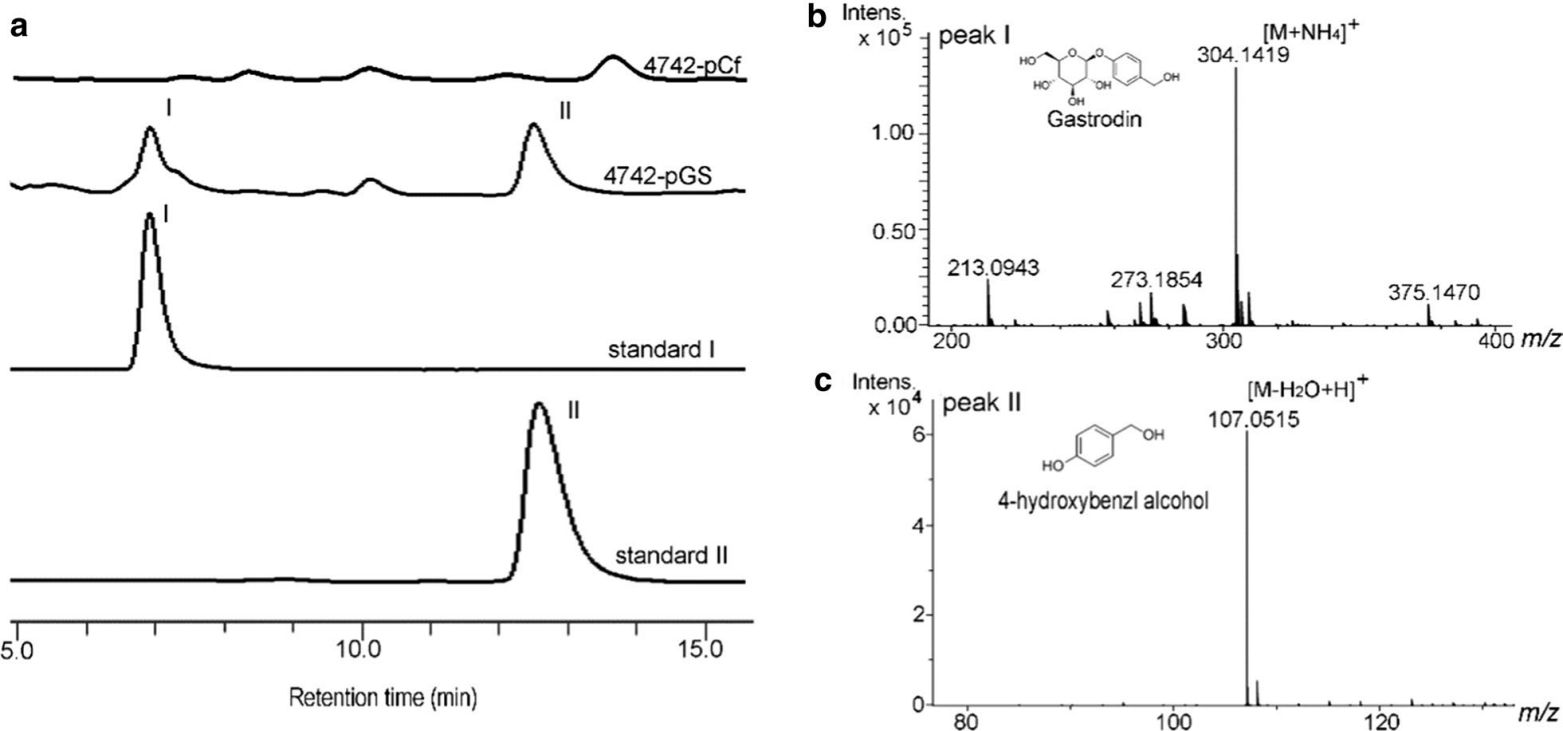
De novo biosynthesis of gastrodin in strain 4742-pGS. **a** HPLC analysis of standard gastrodin (I), standard 4-hydroxybenzyl alcohol (II) and products in the fermentation supernatant of the recombinant strains 4742-pGS (S. *cerevisiae* BY4742 with plasmid pCf302-CP and pCf301-AUA, carrying *CAR*^*syn*^, *PPTcg-1*^*syn*^, *ARO4*^*K229L*^, *ubiC*^*syn*^ and *AsUGT*^*syn*^) and 4742-pCf (control). **b** Mass analysis of peak I from the fermentation supernatant of strain 4742-pGS. **c** Mass analysis of peak II from the fermentation supernatant of strain 4742-pGS

As shown in our previous study, the artificial gastrodin biosynthesis pathway was extended from chorismate [11]. In bacteria, chorismate lyase (UbiC) usually catalyzed chorismate to synthesize 4-HBA and the amounts of 4-HBA always low in cultures [18]. In *S. cerevisiae*, a homolog of UbiC is missing, and the biosynthetic pathway of 4-HBA is unclear [19]. There was no detectable 4-HBA accumulation in *S. cerevisiae* [20]. Overexpression of the feedback inhibition resistance gene *ARO4*^*K229L*^ is usually used to improve the chorismate pathway metabolic flux [14]. In this work, we intended to overexpress *ARO4*^*K229L*^ and UbiC from *E. coli* to preliminarily produce 4-HBA from chorismate in yeast. Coexpression of CAR from the *Nocardia* genus and PPTcg-1 from *C. glutamicum* could effectively reduce protocatechuic acid to protocatechuic aldehyde in *S. cerevisiae* [21]. We tried to overexpress CAR from the *Nocardia* genus and PPTcg-1 from *C. glutamicum* in yeast to realize the reduction of 4-HBA to aldehyde. The subsequent reduction of aldehyde to 4-hydroxybenzylalcohol might be achieved by endogenous alcohol dehydrogenase (ADHs) in yeast. The de novo biosynthetic pathway of gastrodin in *S. cerevisiae* is shown in Fig. 2. We constructed 2 μ-based plasmids carrying strong constitutive promoters to realize target gene overexpression. The plasmid pCf302-CP and the plasmid pCf301-AUA were introduced into *S. cerevisiae* BY4742, and the resulting strain was named 4742-pGS. We observed two clear new compound peaks at retention times of 6.3 min (peak I) and 12.6 min (peak II) in the HPLC spectrum of the fermentation broth supernatant of 4742-pGS compared to that of control 4742-pCf (Fig. 3a). LC–MS analysis showed that peak I had a molecular ion at *m/z* 304.1419 ([M + NH_4_]^+^), which was identical to that of standard gastrodin (Fig. 3b). Peak II had a molecular ion at *m/z* 107.0515 ([M-H_2_O + H]^+^), which was identical to that of standard 4-hydroxybenzyl alcohol (Fig. 3c). The results clearly showed that gastrodin and 4-hydroxybenzylalcohol were produced in strain 4742-pGS, demonstrating that the pathway genes *CAR*^*syn*^, *PPTcg-1*^syn^ and *AsUGT*^*syn*^ were functional in yeast. 4-Hydroxybenzylalcohol was synthesized from 4-HBA by CAR reduction, and subsequently, a portion of the 4-hydroxybenzylalcohol was glycosylated to gastrodin by AsUGT in *S. cerevisiae* BY4742. The overexpression of *ARO4*^*K229L*^ and *E. coli* UbiC might improve the production of 4-HBA from chorismate.

### Gastrodin production was improved by chromosomal integration of biosynthetic pathway genes in the rDNA locus

Stable integration and robust expression of foreign genes is critical for the success of heterologous biosynthesis of valuable compounds. It is challenging to stably express multiple genes in *S. cerevisiae* with the generally used CEN ori-based and 2 µm ori-based plasmids as tools [22, 23]. Moreover, plasmid-based pathway gene expression could result in growth and genetic instability [24]. Chromosomal integration is an ideal method for introducing heterologous genes to achieve robust expression because of its structural stability [25]. The rDNA encompasses approximately 140 copies of tandem repeats on chromosome XII [26], and δ sequences exist in over 100 copies on chromosome XV [27]. The rDNA locus or δ sites are ideal for generating multicopy integration. Some genes associated with natural products, such as ginsenosides [28], glycyrrhetinic acid [29], and salidroside [30], have been successfully integrated into the rDNA locus or δ sites in yeast to achieve multiple-copy and stable expression by different biotechnological methods.

We aimed to simultaneously integrate *CAR*^*syn*^, *PPTcg-1*^*syn*^, *AsUGT*^*syn*^, *ubiC*^*syn*^ and *ARO4*^*K229L*^ into the yeast chromosomal rDNA locus in the expected order by one-step transformation. The arrangement of these genes in the rDNA locus is shown in Fig. 2. In *S. cerevisiae*, the knockout of chorismate mutase (*ARO7*) could block chorismate flux to prephenate and activate the shikimate pathway [20]. Accordingly, we constructed the *aro7*Δ mutant in *S. cerevisiae* BY4742 as the parental strain. First, we PCR-amplified two DNA fragments containing the five target genes and URA3 auxotrophic marker from constructed plasmids in the work. Together with the two fragments rDNA-up and rDNA-down, these DNA fragments (approximately 14 kb) were simultaneously transformed into the strain *S. cerevisiae aro7*Δ by a one-step multiple-fragment yeast transformation protocol. The assembly order and integration of the DNA fragments into the rDNA locus were confirmed by diagnostic PCR and sequencing. Positive colonies were selected and cultured to determine gastrodin production, as shown in Additional file 1:  Figure S1. The resulting strain with the highest concentration of gastrodin was named rGS3.

**Fig. 4:**
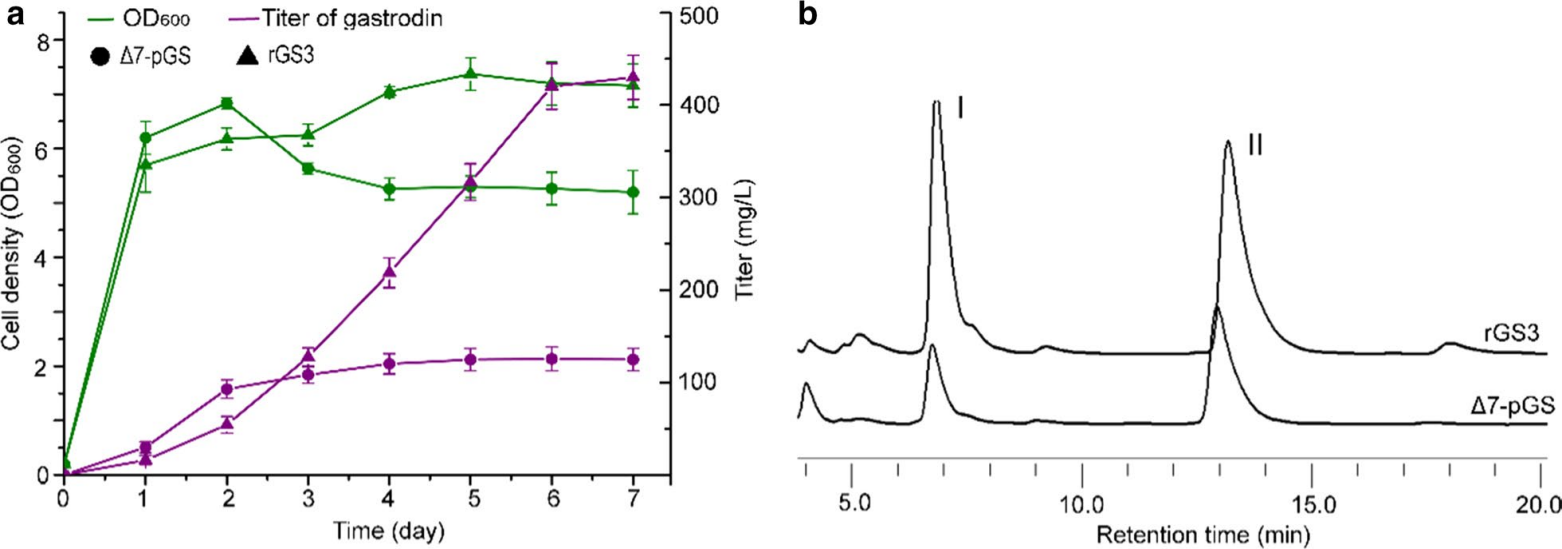
The differential comparison of gastrodin production and growth of strains rGS3 and Δ7-pGS. **a** Time profile of gastrodin production and cell density of strains rGS3 (with pathway genes integrated into rDNA locus) and Δ7-pGS (with pathway genes expressed in plasmids) by flask fermentation. **b** HPLC analysis of gastrodin (I) and 4-hydroxybenzyl alcohol (II) in the fermentation supernatant of strains 7-pGS and rGS3 over 6 days of culture. Three replicates were performed, and the error bars represented standard deviation

Another, we introduced the plasmids pCf302-CP and pCf301-AUA carrying *CAR*^*syn*^, *PPTcg-1*^*syn*^, *AsUGT*^*syn*^, *ubiC*^*syn*^ and *ARO4*^*K229L*^ into *S. cerevisiae aro7*Δ. The generated plasmid-based strain was named Δ7-pGS. Gastrodin production in rGS3 and Δ7-pGS was measured under the same fermentation conditions. Based on the production curves (Fig. 4a), gastrodin production in strain Δ7-pGS reached approximately 120 mg/L and was nearly stable after four days of fermentation. The gastrodin titer of strain rGS3 reached approximately 420 mg/L after 6 days of culture, which was 3.8 times higher than that of the plasmid-based strain Δ7-pGS. HPLC spectra are shown in Fig. 4b. The growth curves showed that there was a growth decline after two days of cultivation of Δ7-pGS, and the OD_600_ of strain rGS3 was 30% higher than that of Δ7-pGS at the end of fermentation (Fig. 4a). These results confirmed that the gene chromosomal integration strategy effectively improved gastrodin production and brought growth recovery compared to plasmid-based pathway gene expression.

### Further improving the precursor supply increased gastrodin production

Enhancement of the precursor supply is a classic and effective strategy to improve final compound production. The initiation of chorismate biosynthesis requires two precursors: erythrose-4-phosphate (E4P) derived from the pentose phosphate pathway and phosphoenolpyruvate (PEP) derived from glycolysis. In *S. cerevisiae*, E4P and PEP were not abundant and were pathway bottlenecks based on the results of carbon tracing and ^13^C metabolic flux analysis [31]*.* Many studies have revealed that the overexpression of transketolase (TktA) and phosphoenolpyruvate synthase *(*PpsA) effectively raised the intracellular pool of E4P and PEP [32, 33]. In yeast, the 3-deoxyD-arabino-heptulosonate-7-phosphate (DAHP) synthase (*ARO4*/*ARO3*) catalyzes the first step to condense PEP and E4P to form DAHP. The pentafunctional enzyme (*ARO1*) catalyzes five subsequent reactions from DAHP to 5-enolpyruvyl-shikimate-3-phosphate (EPSP). Chorismate synthase (*ARO2*) catalyzes the final conversion of EPSP to chorismate. Previous reports showed that co-overexpression of *ARO2*, *ARO1* and *ARO4* (or *ARO3*) could increase AAA-derived aromatic chemical production [15].

**Fig. 5:**
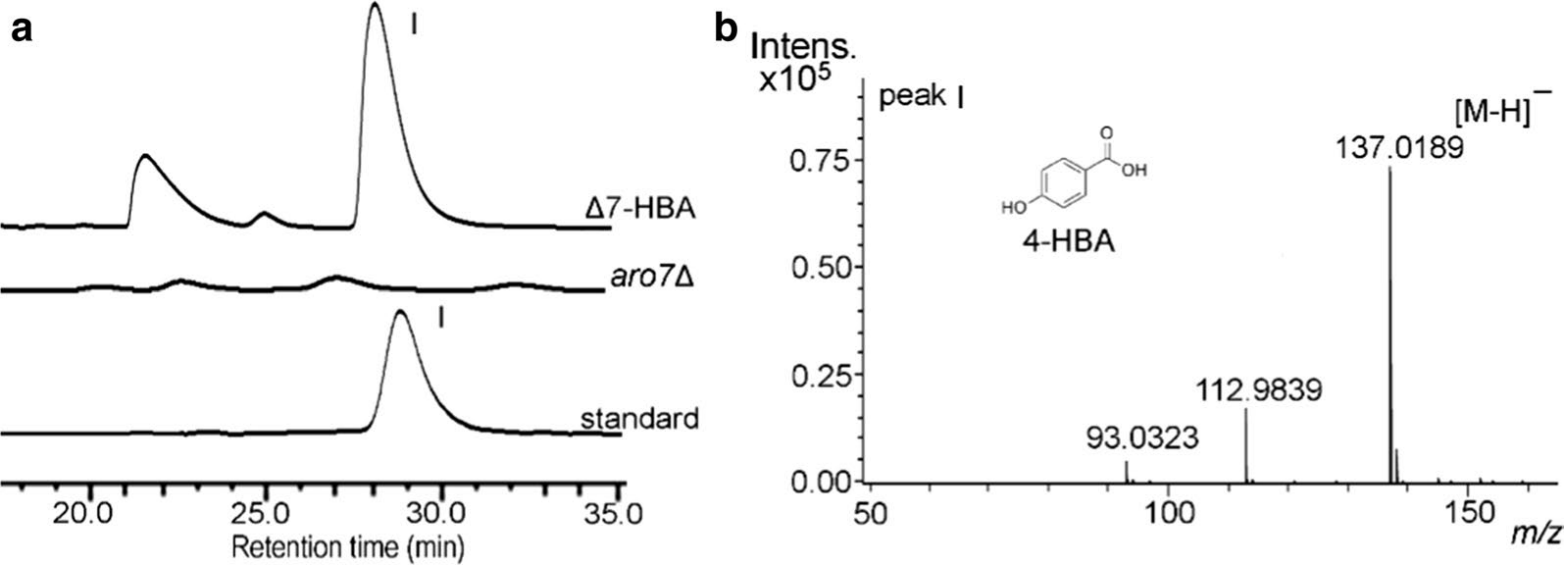
The HPLC analysis and identification of 4-HBA produced in strain Δ7-HBA. **a** HPLC spectrum at 201 nm of the 4-HBA standard and fermentation supernatant of the strains *S. cerevisiae aro7*Δ and Δ7-HBA (with genes of *ARO2*, *ARO1*, *ARO4*^*K229L*^, *ubiC*^*syn*^, *ppsA* and *ktA* integrated into *δ* sites of *S. cerevisiae aro7*Δ). **b** Mass spectrum of peak I in the fermentation supernatant of Δ7-HBA, which was collected and analyzed by LC–MS in negative ion mode in a methanol–water (containing 20 mM NH_4_Ac) gradient system

Given that a deficient supply of precursors might be the cause of the low amount of gastrodin, we intended to further enhance the carbon flux into 4-HBA precursor accumulation by combining the overexpression of *ppsA*, *tktA, ARO1*, and *ARO2* using the multiple-copy integration strategy. First, we PCR-amplified three large DNA fragments containing *ppsA*, *tktA*, *ARO1*, *ARO2*, the mutated *ARO4*^*K229L*^ and *ubiC*^*syn*^ from constructed plasmids in the work. Together with the two fragments *δ* DNA-up and *δ* DNA-down, these DNA fragments (approximately 21 kb) were simultaneously transformed into the strain *S. cerevisiae aro7*Δ by a one-step multiple-fragment yeast transformation protocol. The arrangement of these genes in *δ* sites is shown in Fig. 2. The assembly order and integration of the DNA fragments into the *δ* sites were confirmed by diagnostic PCR and sequencing. Positive colonies were selected and cultured. HPLC analysis of the culture of these colonies showed that there was a clear peak I at a retention time of 28.5 min, similar to the 4-HBA standard, while peak I was absent in *S. cerevisiae aro*7Δ (Fig. 5a). We collected the fraction of peak I for LC–MS in negative ion mode in a methanol–water (containing 20 mM NH_4_Ac) gradient system. LC–MS analysis showed that peak I had a molecular ion at m/z 137.0189 ([M-H]^−^), which was identical to that of standard 4-HBA (Fig. 5b), further confirming 4-HBA overproduction. Production of 4-HBA in some positive colonies was shown in Additional file 1: Figure S2. The colony with the highest 4-HBA production was named Δ7-HBA. Another, consistent with a previous report [20], there was no detectable 4-HBA accumulation in *S. cerevisiae aro7*Δ. These results demonstrated that the combined overexpression of *ARO1*, *ARO2,* mutated *ARO4*^*K229L*^, *ppsA, tktA* and *ubiC*^*syn*^ effectively enhanced metabolic flux toward 4-HBA biosynthesis in yeast.

**Fig. 6:**
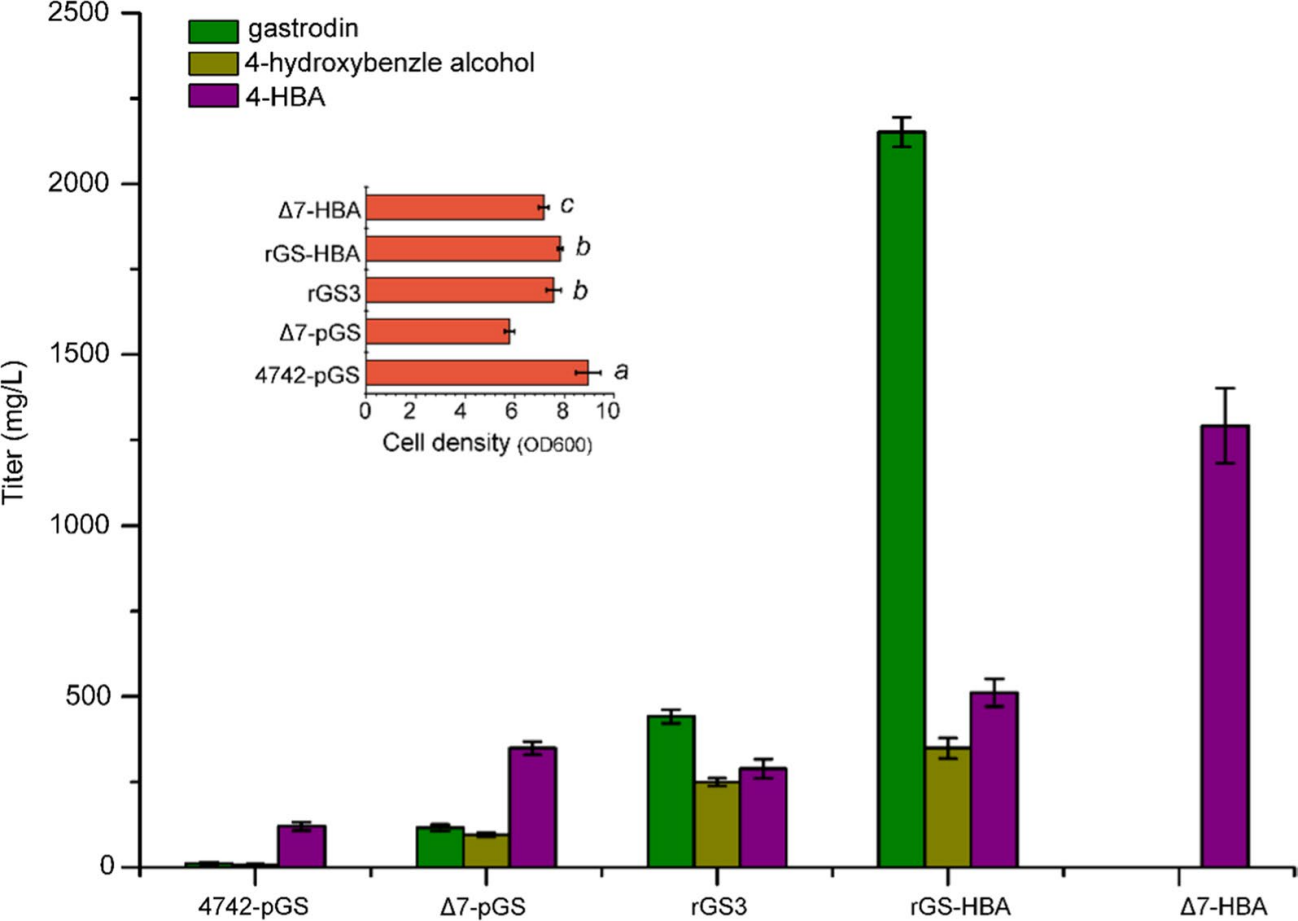
Titers of gastrodin, 4-hydroxybenzyl alcohol and 4-HBA, and the cell density of engineered strains. The strain 4742-pGS, S. *cerevisiae* BY4742 with plasmid pCf302-CP and pCf301-AUA (carrying *CAR*^*syn*^, *PPTcg-1*^*syn*^, *ARO4*^*K229L*^, *ubiC*^*syn*^ and *AsUGT*^*syn*^); the strain Δ7-pGS, *S. cerevisiae aro7*Δ with plasmids pCf302-CP and pCf301-AUA (carrying *CAR*^*syn*^, *PPTcg-1*^*syn*^, *ARO4*^*K229L*^, *ubiC*^*syn*^ and *AsUGT*^*syn*^); the strain rGS3, with genes of *CAR*^*syn*^, *PPTcg-1*^*syn*^, *ARO4*^*K229L*^, *ubiC*^*syn*^ and *AsUGT*^*syn*^ integrated into rDNA locus of *S. cerevisiae aro7*Δ; the strain Δ7-HBA, with genes of *ARO2*, *ARO1*, *ARO4*^*K229L*^, *ubiC*^*syn*^, *ppsA* and *ktA* integrated into *δ* sites of *S. cerevisiae aro7*Δ; the strain rGS-HBA with genes of *ARO2*, *ARO1*, *ARO4*^*K229L*^, *ubiC*^*syn*^, *ppsA* and *ktA* integrated into *δ* sites of strain rGS3. Three replicates were performed, and the error bars represented standard deviation. The significance differences of cell density between the control Δ7-pGS and other engineered strain were analyzed by Student’s t-test. a, p-value < 0.01; b, p-value < 0.05; c, no statistical significance differences

Subsequently, these five DNA fragments were transformed into *S. cerevisiae* rGS3. Gastrodin production in the positive colonies was measured (Additional file 1: Figure S3), and the colony with the highest gastrodin production was named rGS-HBA. To evaluate the product titers and cell density of the engineered strains, we carried out fermentation in mineral medium with glucose as the sole carbon source by a 250-mL flask fermentation. The titers of gastrodin, 4-hydroxybenzyl alcohol and 4-HBA were determined after 6 days of fermentation. As shown in Fig. 6, the original gastrodin-producing strain 4742-pGS had 12 mg/L gastrodin, accumulated 8 mg/L 4-hydroxybenzyl alcohol and 120 mg/L 4-HBA. In strain 4742-pGS, the accumulation of 4-HBA showed that the overexpression of ARO4 ^K229L^ and *E. coli* UbiC indeed enhanced 4-HBA production. Compared to 4742-pGS, the production of all the three compounds was increased in Δ7-pGS, indicating that deletion of *ARO7* activated the shikimate pathway and promoted the biosynthesis of gastrodin, 4-hydroxybenzyl alcohol and 4-HBA. The cell density of Δ7-pGS (OD_600_ = 5.8) was lower than that of 4742-pGS (OD_600_ = 9.0), probably because deletion of *ARO7* affected strain growth. The plasmid-free strains rGS, Δ7-HBA and rGS-HBA showed similar cell densities, higher than that of plasmid-based Δ7-pGS. In addition to gastrodin, the total amount of 4-hydroxybenzylalcohol produced by plasmid-free rGS3 was 3 times that produced by the plasmid-based strain Δ7-pGS. These results further confirmed that the genes integrating expression in yeast were superior to plasmid-based expression in gastrodin production. With the cooverexpression of *ppsA*, *tktA*, *ARO1*, *ARO2*, *ARO4*^*K229L*^ and *ubiC*^*syn*^ integrated in *δ* sites, 1.3 g/L 4-HBA accumulation was observed in Δ7-HBA. The enhanced pool of 4-HBA further increased the metabolic flux toward the aglycon 4-hydroxybenzyl alcohol, and consequently, high gastrodin production (2.1 g/L) was achieved in strain rGS-HBA, which was approximately 4.8 times that of *S. cerevisiae* rGS. With these rational engineering, the titer of gastrodin was improved 175 times higher than that of the original 4742-pGS.

The expression levels of target genes were detected between engineered strains by quantitative real-time PCR. Relative transcript levels were analyzed individually after normalization to the actin internal reference gene. Results were as shown in Additional file 1: File S1The  analysis of expression levels of target genes. The changes of transcript expression levels of target genes showed good consistency with the production of gastrodin between engineered strains.

## Conclusions

Here, we explored the potential of *S. cerevisiae* as the host for high-level production of gastrodin from glucose. The glucosyltransferase AsUGT was firstly identified to convert 4-hydroxybenzylalcohol to gastrodin with high catalytic efficiency in yeast. The de novo biosynthesis of gastrodin from glucose was realized by introducing the *AsUGT*^*syn*^ and other pathway genes into *S. cerevisiae*. With multiple-copy integration of the entire pathway genes in the rDNA locus and enhancement of metabolic flux toward 4-hydroxybenzyl alcohol, the best optimized strain produced 2.1 g/L gastrodin in mineral medium with glucose as the sole carbon source. To our knowledge, this is the highest reported titer of gastrodin produced by an engineered microorganism. Instead of chemical synthesis or plants extraction, this work provides a strong basis for the biotechnological production of gastrodin at an industrial scale for pharmaceutical applications.

## Material and methods

### Strains and medium

All plasmids and strains used in this work are shown in Table 1. *E. coli* DH5α was used for plasmid construction. *E. coli* strains were cultivated in Luria broth (LB). *S. cerevisiae* BY4742 (*MATα his3*Δ1 *leu2*Δ0 *lys2*Δ0 *ura3*Δ0) was used as the parental strain for all the yeast strains. Yeast strains were grown in SC dropout medium containing 6.7 g/L yeast nitrogen base, 2 g/L dropout mix, and 20 g/L glucose. SC dropout medium without uracil (SC-ura), SC dropout medium without leucine (SC-leu), and SC dropout medium without uracil and leucine (SC-ura, leu) were used in this work. Yeast strains were also cultured in YPD medium containing 10 g/L yeast extract, 20 g/L peptone and 20 g/L glucose. Standards, including gastrodin, 4-hydroxybenzl alcohol and 4-HBA, were purchased from Aladdin Chemistry Co., Ltd. (Shanghai, China).

**Table 1:** Strains and plasmids used in this study

	Description	Reference
Strains		
* S. cerevisiae* BY4742	*MATα*; *his3*Δ1; *leu2*Δ0; *lys2*Δ0; *ura3*Δ0	Novagen
4742–301	BY4742 with pCf301	This study
4742-UGT73B6^FSy^	BY4742 with pCf301-*UGT73B6*^*FSy*^	This study
4742-AsUGT^syn^	BY4742 with pCf301-*AsUGT*^*syn*^	This study
4742-pCf	BY4742 with pCf301& pCf302	This study
4742-pGS	BY4742 with pCf302-CP & pCf301-AUA	This study
* S. cerevisiae aro7*Δ	The deletion of *ARO7* in *S. cerevisiae* BY4742	This study
Δaro7-pCf	*S. cerevisiae aro7*Δ with pCf301 & pCf302	This study
Δ7-pGS	*S. cerevisiae aro7*Δ with pCf302-CP & pCf301-AUA	This study
rGS3	P_TDH3_-*PPTcg-1*^*syn*^-T_TEF_-P_ScTEF1_-*CAR*^*syn*^-T_CYC1_, *URA3*-T_ADH1_-*ARO4*^*K229L*^-P_PGK1_-P_TDH3_-*ubiC*^*syn*^-T_TEF_-P_ScTEF1_-*AsUGT*^*syn*^-T_CYC1_ cassettes were integrated into rDNA locus of *S. cerevisiae aro7*Δ	This study
Δ7-HBA	P_TDH3_-*ARO2*-T_TEF_-P_ScTEF1_-*ARO1*-T_CYC1_, *LEU2*-T_ADH1_-*ARO4*^*K229L*^-P_PGK1_-P_TDH3_-*ubiC*^*syn*^-T_TEF_, P_TDH3_-*ppsA*-T_TEF_-P_ScTEF1_-*tktA*-T_CYC1_ cassettes were integrated into *δ* sites of *S. cerevisiae aro7*Δ	This study
rGS-HBA	P_TDH3_-*ARO2*-T_TEF_-P_ScTEF1_-*ARO1*-T_CYC1_, *LEU2*-T_ADH1_-*ARO4*^*K229L*^-P_PGK1_-P_TDH3_-*ubiC*^*syn*^-T_TEF_, P_TDH3_-*ppsA*-T_TEF_-P_ScTEF1_-*tktA* -T_CYC1_ cassettes were integrated into *δ* sites of rGS3	This study
Plasmids		
pCf301	URA3; 2 μm ori with P_PGK1_, P_TDH3_, P_TEF1_; *Amp*	In our lab ^30^
pCf302	LEU2; 2 μm ori with P_PGK1_, P_TDH3_, P_TEF1_; *Amp*	In our lab ^30^
pCf301-UGT73B6^FSy^	pCf301 carrying P_ScTEF1_-*UGT73B6*^*FSy*^-T_CYC1_	This study
pCf301-* AsUGT*^*syn*^	pCf301 carrying P_ScTEF1_-*AsUGT*^*syn*^-T_CYC1_	This study
pCf302-CP	pCf302 carrying P_ScTEF1_-*CAR*^*syn*^ -T_CYC1_, P_TDH3_-*PPTcg-1*^*syn*^ -T_TEF_	This study
pCf301-AUA	pCf301 carrying P_PGK1_-*ARO4*^*K229L*^-T_ADH1_, P_TDH3_-*ubiC*^*syn*^ -T_TEF_, P_ScTEF1_-*AsUGT*^*syn*^-T_CYC1_	This study
pCf302-AU	pCf302 carrying P_PGK1_-*ARO4*^*K229L*^-T_ADH1_, P_TDH3_-*ubiC*^*syn*^ -T_TEF_	This study
pCf301-pt	pCf301 carrying P_TDH3_-*pps *-T_TEF_-P_ScTEF1_-*tktA*-T_CYC1_	This study
pCf301-*ARO1/2*	pCf301 carrying P_TDH3_-*ARO*-T_TEF_-P_ScTEF1_-*ARO1*-T_CYC1_	This study

### Genes and enzymes

Glucosyltransferase *AsUGT* (Q9AR73) [17] from *Rauvolfia serpentina* (serpentwood), Rhodiola-derived glycosyltransferase UGT73B6 mutant gene *UGT73B6*^*FS*^ [11], carboxylic acid reductase CAR (Q6RKB1.1) gene [21] from *Nocardia iowensis*, phosphopantetheinyl transferase PPTcg-1 (WP_003857486.1) gene [21] from *Corynebacterium glutamicum*, and chorismate pyruvate-lyase UbiC (AAY88959.1) [34] gene from *E. coli* BL21 (DE3) were synthesized with *S. cerevisiae* codon optimization by Generay Biotech Co., Ltd. (Shanghai, China). The feedback-resistant DAHP synthase mutant ARO4^K229L^ from *S. cerevisiae* was generated from DAHP synthase *ARO4* (NP_009808) by a site-directed mutagenesis method. The pentafunctional EPSP synthase gene *ARO1* (Gene ID: 851705) and chorismate synthase gene *ARO2* (GeneID: 852729) were amplified from *S. cerevisiae* BY4742. The phosphoenolpyruvate synthase gene *ppsA* (Gene ID: 946209) and transketolase gene *tktA* (Gene ID: 947420) were amplified from *E. coli* MG1655. The synthesized genes and sequences applied in this study are showed in Additional file 1: Table S2.

### Vector construction

All the primers used in this study are listed in Additional file 1: Table S1.

The codon-optimized genes *AsUGT*^*syn*^ and *UGT73B6*^*FSy*^ in *S. cerevisiae* were separately cloned into the pCf301 [30] vector using *Nhe*I/*Aat*II to create the plasmids pCf301- *AsUGT*^*syn*^ and pCf301- *UGT73B6*^*FSy*^, respectively. The codon-optimized genes *CAR*^*syn*^ and *PPTcg-1*^*syn*^ were respectively cloned into the pCf302 [30] vector using *Nhe*I*/Aat*II and *Hind*III*/Bam*HI to create the pCf302-CP plasmids. The *ARO4*^*K229L*^, codon-optimized *AsUGT*^*syn*^, and *ubiC*^*syn*^ genes were respectively cloned into the pCf301 vector using *Spe*I*/Bgl*II, *Nhe*I*/Aat*II and *Hind*III*/Bam*HI to create the pCf301-AUA plasmids. The genes *ARO4*^*K229L*^ and *ubiC*^*syn*^ were also respectively cloned into the pCf302 vector using *Spe*I*/Bgl*II and *Hind*III*/Bam*HI to create the pCf302-AU plasmids. The *ppsA* and *tktA* genes were respectively cloned into the pCf301 vector using *Nhe*I*/Aat*II and *Hind*III*/Bam*HI to create the pCf301-pt plasmids. The genes *ARO1* and *ARO2* were respectively cloned into the pCf301 vector using *Nhe*I*/Aat*II and *Hind*III*/Bam*HI to create the pCf301-ARO1/2 plasmids.

### Chromosomal disruption of *ARO7*

Targeted chromosomal disruption of the chorismate mutase gene *ARO7* in the strain *S. cerevisiae* BY4742 was performed by homologous recombination using antibiotic marker-containing disruption cassettes created by PCR, as described by Gueldener et al*.* [35]. The *loxP*-flanked *kanMX* cassettes were amplified from pUG6. The antibiotic marker was removed using the Cre recombinase plasmid pSH65. Clones successfully carrying the *ARO7* disruption cassette were further confirmed by colony PCR and named *S. cerevisiae* BY4742 *aro7*Δ.

### Feeding experiments

The *S. cerevisiae* BY4742 strains respectively harboring pCf301, pCf301-*UGT73B6 *^*FSy*^ or pCf301-*AsUGT*^*syn*^ were cultured in 3 mL of SC-ura medium at 30 °C and 220 r.p.m. for 24 h. Then, 2 mL of the culture was diluted into 50 mL of medium and shaken at 30 °C at 220 r.p.m. for 18 h. Then, the substrate 4-hydroxybenzyl alcohol was added into the cultures at a final concentration of 2 mM. After 24 h and 48 h, samples were taken and analyzed by HPLC or HPLC–MS. Three replicates were performed in experiments. The data were presented as means ± SD (standard deviation).

### Yeast cultivation

All measurements of optical density at 600 nm (OD_600_) were made using a SHIMADZU UV-1800 spectrophotometer. A single colony was first inoculated into 35-mL culture tubes containing 3 mL of SC dropout medium and grown at 30 °C and 220 r.p.m. for 18 h. Then, seed cultures were transferred into 50 mL of SC dropout medium to obtain an OD_600_ of 0.2 in 250-mL culture flasks. The strains were cultured at 30 °C and 220 r.p.m. for approximately 7 days. Samples were taken every 24 h and analyzed by HPLC. All of the data generated in this study were collected from three independent experiments. The data were presented as means ± SD (standard deviation).

### One-step multiple-fragment yeast transformation

The procedure of multiple-fragment transformation was based on the method described by Jolanda van Leeuwen for rapid and efficient plasmid construction [36]. A single colony was inoculated into 3 mL of liquid medium and incubated overnight at 200 r.p.m. and 30 °C. Then, 2 mL of the overnight culture was diluted into 50 mL of medium to obtain an OD_600_ of 0.2 and incubated at 30 °C and 200 r.p.m. The culture with an OD_600_ of 0.8 was centrifuged at 2000*g* for 3 min, and the cell pellet was suspended in 25 mL of sterile water. After centrifugation, the cells were suspended in 1.0 mL of 0.1 M lithium acetate (LiAc). The cell suspension was transferred to a 1.5-mL microcentrifuge tube and centrifuged for 15 s at 2000*g*. After discarding the supernatant, the cells were suspended in 320 μL of LiAc (0.1 M) as competent cells. Ten microliters of DNA fragment mixture (100–200 ng of each DNA fragment), 2 µL of ssDNA (10 mg/mL) and 12 µL of competent yeast cells were added into a 1.5-mL microcentrifuge tube and mixed gently. Then, 100 µL of the transformation buffer (800 µL of 50% PEG3350, 100 µL of 1 M LiAc, 100 µL of 10 × TE, and 50 µL of DMSO) was added into the 1.5-mL microcentrifuge tube and vortexed for 10 s. After incubation for 30 min at 25 °C, the transformation mixture was incubated at 42 °C for 15 min and then on ice for 5 min. The transformation mixture was centrifuged at 800*g* for 1 min, and the cells were suspended in 1 mL of YPD. The cell suspension was incubated at 30 °C for 2 h and was centrifuged at 2000*g* for 1 min. The cells were suspended in sterile water and plated on SC plates lacking leucine and/or uracil. Plasmid transformation was carried out using the standard LiAc method.

### Chromosomal multiple-copy integration of the pathway genes for gastrodin biosynthesis

The *CAR*^*syn*^, *PPTcg-1*^*syn*^, *AsUGT*^*syn*^, *ubiC*^*syn*^ and *ARO4*^*K229L*^, *URA*3 selectable marker, a total sequence of approximately 14 kb, were integrated into the ribosomal DNA (rDNA) locus. The two fragments rDNA-up (476 bp) and rDNA-down (326 bp) were PCR-amplified from the DNA of *S. cerevisiae* BY4742 using the primer set rU-F&R and rD-F&R. An approximately 5.8-kb fragment containing *CAR*^*syn*^ and *PPTcg-1*^*syn*^ was PCR-amplified from the pCf302-CP plasmids using the primer set r5.8-F & R. An approximately 7.4-kb fragment containing *AsUGT*^*syn*^, *ubiC*^*syn*^*, ARO4*^*K229L*^ and *URA3* was PCR-amplified from the pCf301-AUA plasmids using the primer set r7.4-F & R. The four DNA fragments, with 30–40 bp homologous regions designed between the adjacent fragments, were transferred into *S. cerevisiae aro7*Δ using the protocol for one-step multiple-fragment transformation mentioned above. The colonies were selected on the SC-ura plates and verified by PCR amplification and sequencing. Positive colonies were cultured, and the highest gastrodin-producing strain was designated rGS3.

The *ARO1*, *ARO2, ppsA*, *tktA*, *ubiC*^*syn*^, *ARO4*^*K229L*^ and *LEU2* selectable marker, a total sequence length of approximately 21 kb, were integrated separately into the *δ*-sites of the strains *S. cerevisiae aro7*Δ and rGS3. The two fragments *δ* DNA-up (329 bp) and *δ* DNA-down (316 bp) were PCR-amplified from the DNA of *S. cerevisiae* BY4742 using the primer set *δ* U-F&R and *δ* D-F&R. A 7.7-kb fragment containing *ARO1* and *ARO2* was PCR-amplified from the pCf301-*ARO1/2* plasmid using the primer set *δ* 7.7-F&R. An approximately 6.8-kb fragment containing *LEU2*, *ubiC*^*syn*^*,* and *ARO4*^*K229L*^ was PCR-amplified from the pCf302-AU plasmid using the primer set *δ* 6.8-F&R. A 6.2-kb fragment containing *ppsA* and *tktA* was amplified from the pCf301-pt plasmid using the primer set *δ* 6.2-F&R. The five DNA fragments, with 30–40 bp homologous regions designed between the adjacent fragments, were transformed into the strain *S. cerevisiae aro7*Δ using the protocol for one-step multiple-fragment transformation mentioned above. Positive colonies were selected on the SC-leu plates, and the strain with the highest 4-HBA production was designated strain Δ7-HBA. The five DNA fragments were also transformed into the strain rGS3. Colonies were selected on the SC-ura, leu plates, and the strain with the highest gastrodin production was designated strain rGS-HBA. The sequences of the rDNA and *δ* DNA fragments applied are shown in Additional file 1: Table S2.

### Metabolite analysis

The chemicals gastrodin, 4-hydroxybenzl alcohol and 4-HBA were analyzed by HPLC on a Shimadzu system with a UV detector. The column used was an Agela Innoval C18, 4.6 × 250 mm, with a particle size of 5 μm. A methanol–water (containing 0.1% trifluoroacetic acid) gradient system at a flow rate of 1 mL/min was used to separate the compounds. The HPLC program was as follows: 10% methanol for 40 min, 10% to 100% methanol for 1 min, 100% methanol for 10 min, 100% to 10% methanol for 1 min, and 10% methanol for 10 min. Products were detected at 224 nm or 201 nm. HPLC–MS was performed on an Agilent 1260 system with a 1260 Infinity UV detector and a Bruker micro Q-TOF II mass spectrometer equipped with an ESI ionization probe. The HPLC conditions were the same as described above. For HPLC–MS analysis of 4-HBA, the fraction of the peak was collected for MS identification in negative ion mode, and methanol–water (containing 20 mM NH_4_Ac) was used as the gradient system. The other conditions were the same as described above. To quantify the titers of gastrodin, 4-hydroxybenzl alcohol and 4-HBA, standard calibration curves were prepared with a series of known concentrations of the standard. R2 value for the standard curve was higher than 0.999. Samples were diluted as required for falling into the concentration range of the calibration curves. The titers were presented as means ± SD (standard deviation).

## RNA isolation and quantitative real-time PCR

Total RNA from *S. cerevisiae* was isolated and purified using hot-acid-phenol method. To synthesize cDNA, about 2.0 μg of RNA was added to a FastKing gDNA Dispelling RT SuperMix (TIANGEN Biotech, Beijing, China). Each 20.0 μL reaction contained 10.0 μL of SuperReal PreMix Plus (SYBR green) (TIANGEN Biotech, Beijing, China). Real-time PCR was monitored and analyzed using Roche LightCycler^®^ 96 System. Samples of Δ7-pGS and rGS3 were prepared after 2 days and 4 days fermentation culture. Samples of rGS3-PHB were prepared after 2 days. All samples were prepared in triplicate. The relative expression level of genes in engineered strain was calculated by the relative quantitative 2^−ΔΔCT^ method [37] and ß-actin was used as the internal control gene Additional file 1: File S1.

## Supplementary information

**Additional files. File S1. ** The analysis of expression levels of target genes. **Figure S1. **Titer of gastrodin produced by colonies with gene integration into the yeast chromosomal rDNA locus. **Figure S2.** Titer of 4-HBA accumulated in the culture of colonies with gene integration into yeast chromosomal *δ* sites. **Figure S3.** Titer of gastrodin produced by colonies with gene integration into yeast chromosomal *δ* sites. **Figure S4.** The relative transcript levels of target genes in rGS3 compared to Δ7-pGS at 2 day and 4 day. **Figure S5.** The relative transcript levels of target genes in rGS3-PHB compared to rGS3 at 2 day. **Table S1.** The primers used in this study. **Table S2.** The synthesized genes, sequence of rDNA and *δ* DNA fragment applied in this study. **Table S3.** The real-time PCR primers used in this study. **Table S4.** Information of genes used in this study.

## Data Availability

All data generated or analyzed during this study are included in this article and its Additional file 1.
