# Correction to: Metabolic engineering of *Saccharomyces cerevisiae* for high-level production of gastrodin from glucose

**DOI:** 10.1186/s12934-022-01747-y

**Published:** 2022-02-04

**Authors:** Hua Yin, Tiandong Hu, Yibin Zhuang, Tao Liu

**Affiliations:** 1 Tianjin Institute of Industrial Biotechnology, Chinese Academy of Sciences, Tianjin, 300308 China; 2 Key Laboratory of Systems Microbial Biotechnology, Chinese Academy of Sciences, Tianjin, 300308 China

## Correction to: Microb Cell Fact (2020) 19:218 https://doi.org/10.1186/s12934-020-01476-0

Following publication of the original article [1], the authors identified an error in acknowledgement section. The correct acknowledgement is given below.

- This work was supported by grants from the National Natural Science Foundation of China (**31770104**, 31970065, U1902214).
