# Supplementary material for: Metabolic engineering of Saccharomyces cerevisiae for high-level production of gastrodin from glucose
Source: Microb Cell Fact. 2020 Nov 26;19:218. doi: 10.1186/s12934-020-01476-0 (PMC7690157; doi:10.1186/s12934-020-01476-0)
Supplement: Supplementary file 1 — Additional files. File S1. The analysis of expression levels of target genes. Figure S1. Titer of gastrodin produced by colonies with gene integration into the yeast chromosomal rDNA locus. Figure S2. Titer of 4-HBA accumulated in the culture of colonies with gene integration into yeast chromosomal δ sites. Figure S3. Titer of gastrodin produced by colonies with gene integration into yeast chromosomal δ sites. Figure S4. The relative transcript levels of target genes in rGS3 compared to Δ7-pGS at 2 day and 4 day. Figure S5. The relative transcript levels of target genes in rGS3-PHB compared to rGS3 at 2 day. Table S1. The primers used in this study. Table S2. The synthesized genes, sequence of rDNA and δ DNA fragment applied in this study. Table S3. The real-time PCR primers used in this study. Table S4. Information of genes used in this study. [file 12934_2020_1476_MOESM1_ESM.docx]

**Supplementary information**

**Metabolic Engineering of *Saccharomyces cerevisiae* for High-Level Production of Gastrodin from Glucose**

**Hua Yin ^1, 2^, Tiandong Hu^1, 2^, Yibin Zhuang^1, 2^, Tao Liu ^1, 2,^ ***

^1^ Tianjin Institute of Industrial Biotechnology, Chinese Academy of Sciences, Tianjin 300308, China

^2^ Key Laboratory of Systems Microbial Biotechnology, Chinese Academy of Sciences, Tianjin 300308, China

Address correspondence to Tao Liu, [liu_t@tib.cas.cn](mailto:liu_t@tib.cas.cn)

**Table of Contents**

**File S1: The analysis of expression levels of target genes.**

**Figure S1** Titer of gastrodin produced by colonies with genes integrated into the yeast chromosomal rDNA locus.

**Figure S2** Titer of 4-HBA accumulated in the culture of colonies with genes integrated into yeast chromosomal *δ* sites.

**Figure S3** Titer of gastrodin produced by colonies with genes integrated into yeast chromosomal *δ* sites.

**Figure S4** The relative transcript levels of target genes in rGS3 compared to Δ7-pGS at 2 day and 4 day.

**Figure S5** The relative transcript levels of target genes in rGS3-PHB compared to rGS3 at 2 day.

**Table S1** The primers used in this study.

**Table S2** The synthesized genes, sequence of rDNA and *δ* DNA fragments applied in this study.

**Table S3** The real-time PCR primers used in this study.

**Table S4** Information of genes used in this study.

**File S1: The analysis of expression levels of target genes**

The expression levels of target genes were detected between Δ7-pGS and rGS3, rGS3 and rGS3-PHB by quantitative real-time PCR. Relative transcript levels were analyzed individually after normalization to the actin internal reference gene. As shown in Figure S4, the relative transcript levels of the 3.5 kb *CAR^syn^* gene had over twenty folds increase in rGS3 relative to Δ7-pGS at 2 day and 4 day. The relative transcript levels of *ubiC^syn^* and *ARO4* showed about 19.9 and 3.9 times increase respectively in rGS3 relative to Δ7-pGS at 4 day. The results were consistent with the gastrodin production of rGS3 exceeding Δ7-pGS after 4 days fermentation culture. As shown in Figure S5, the relative expression levels of genes *ubiC^syn^, ARO4,* *ARO1* and *ARO2* had over two folds increase in rGS3-PHB compared to rGS3 (Figure S5). The mean CT of the ß-actin reference gene in rGS3-PHB and rGS3 was 21.5 and 20.8, respectively. The mean CT of the *ppsA* and *tktA* gene in rGS3 respectively was 36.3 and 37.2, which were beyond 35. The mean CT of the *ppsA* and *tktA* gene in rGS3-PHB respectively was 31.8 and 25.9. These results suggested that *ppsA* and *tktA* genes had no expression in rGS3 and were expressed at transcript level in rGS3-PHB.The *δ* sites integration of *ARO1*, *ARO2*, *ARO4^K229L^*, *ubiC^syn^*, *ppsA* and *tktA* enhanced their transcript levels in rGS3-PHB.


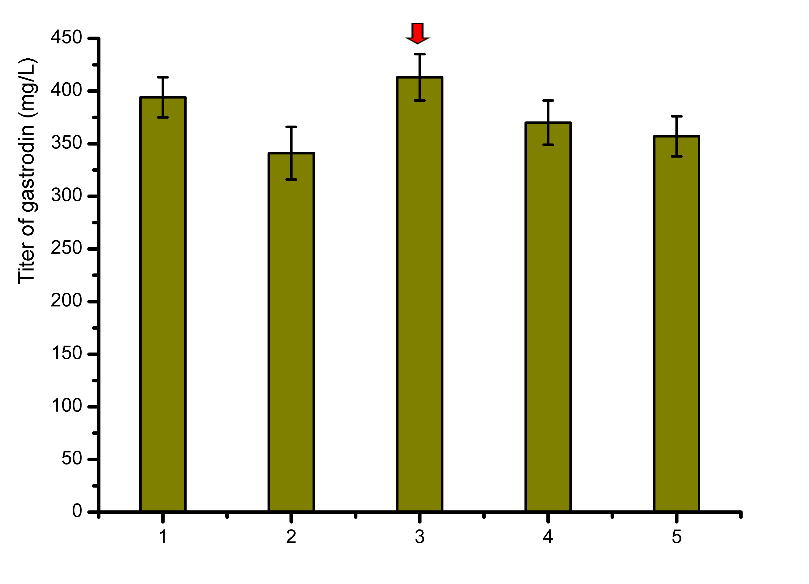


**Figure S1**. Titer of gastrodin produced by colonies with genes integraed into the yeast chromosomal rDNA locus. The genes, including *CAR^syn^*, *PPTcg-1^syn^*, *AsUGT^syn^*, *ubiC^syn^* and *ARO4^K229L^,* were integrated into the rDNA locus of *S. cerevisiae aro7*Δ, and the resulting strain with the highest titer was designated rGS3 (indicated with a red arrow). The error bars represented standard deviation.


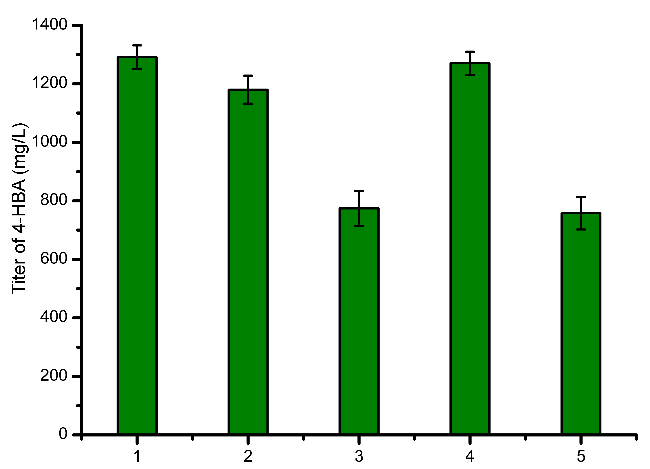


**Figure S2**. Titer of 4-HBA accumulated in the culture of colonies with genes integrated into yeast chromosomal *δ* sites. The genes, including *ARO1*, *ARO2ppsA*, *tktA*, *ubiC^syn^* and *ARO4^K229L^*, were integrated into *δ* sites of *S. cerevisiae* *aro7*Δ. The error bars represented standard deviation.


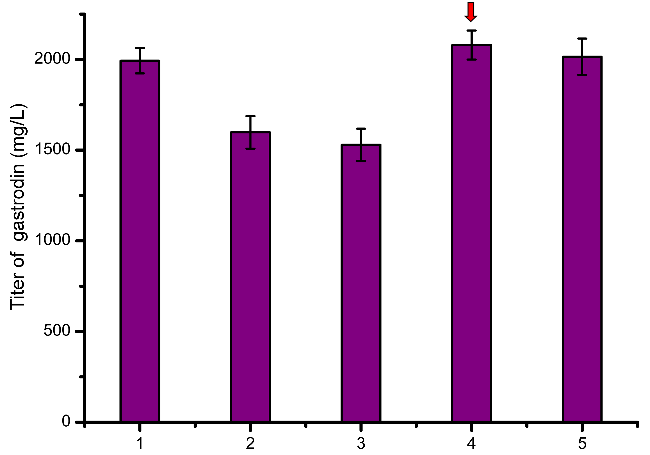


**Figure S3**. Titer of gastrodin produced by colonies with genes integrated into yeast chromosomal *δ* sites. The genes, including *ARO1*, *ARO2, ppsA*, *tktA*, *ubiC^syn^* and *ARO4^K229L^*, were integrated into *δ* sites of rGS3, and the resulting strain with the highest titer was designated rGS-HBA (indicated with a red arrow). The error bars represented standard deviation.


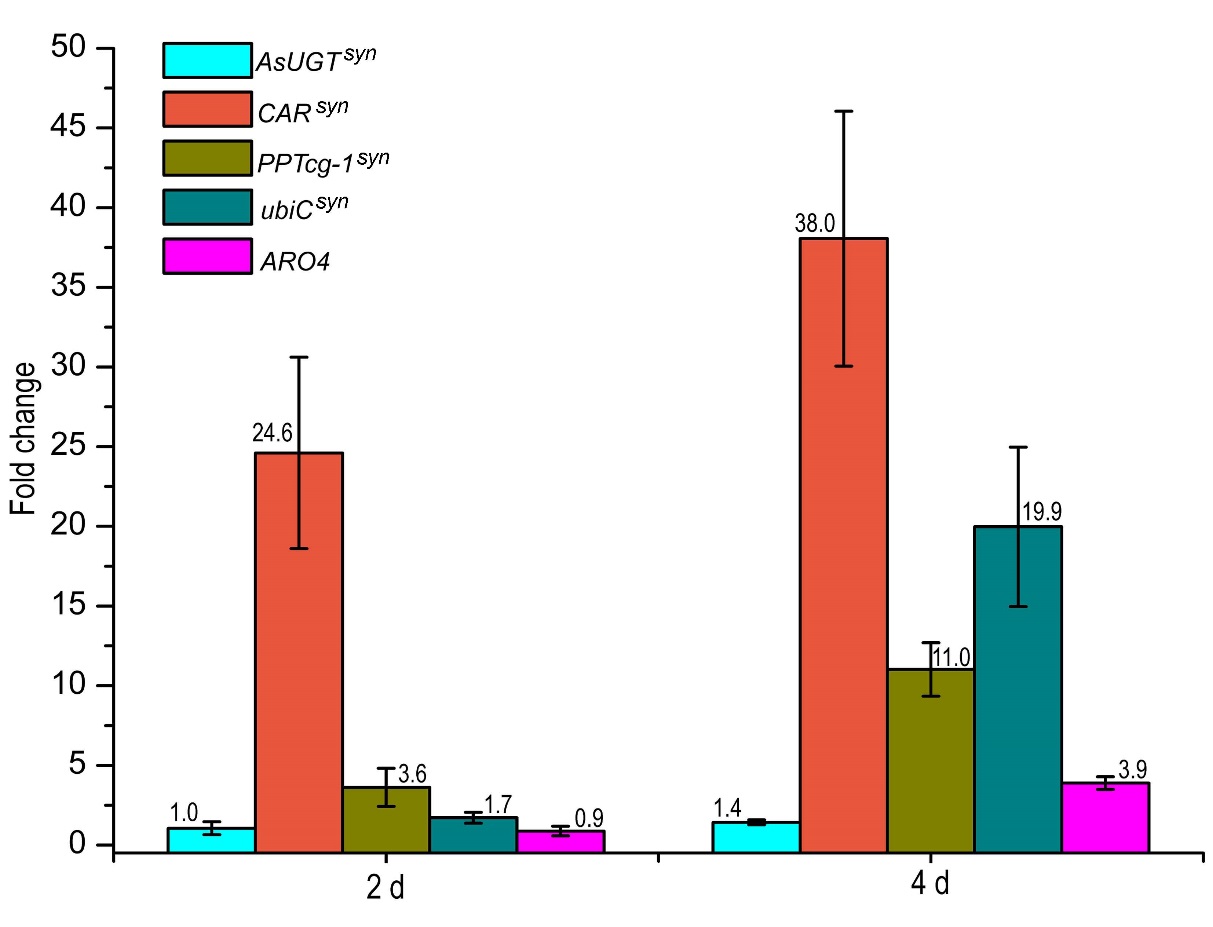


**Figure S4**. The relative transcript levels of target genes in rGS3 compared to Δ7-pGS at 2 day and 4 day. The fold changes were calculated by the relative quantitative 2^−ΔΔCt^ method and actin was used as the internal standard. The error bars represented standard deviation.


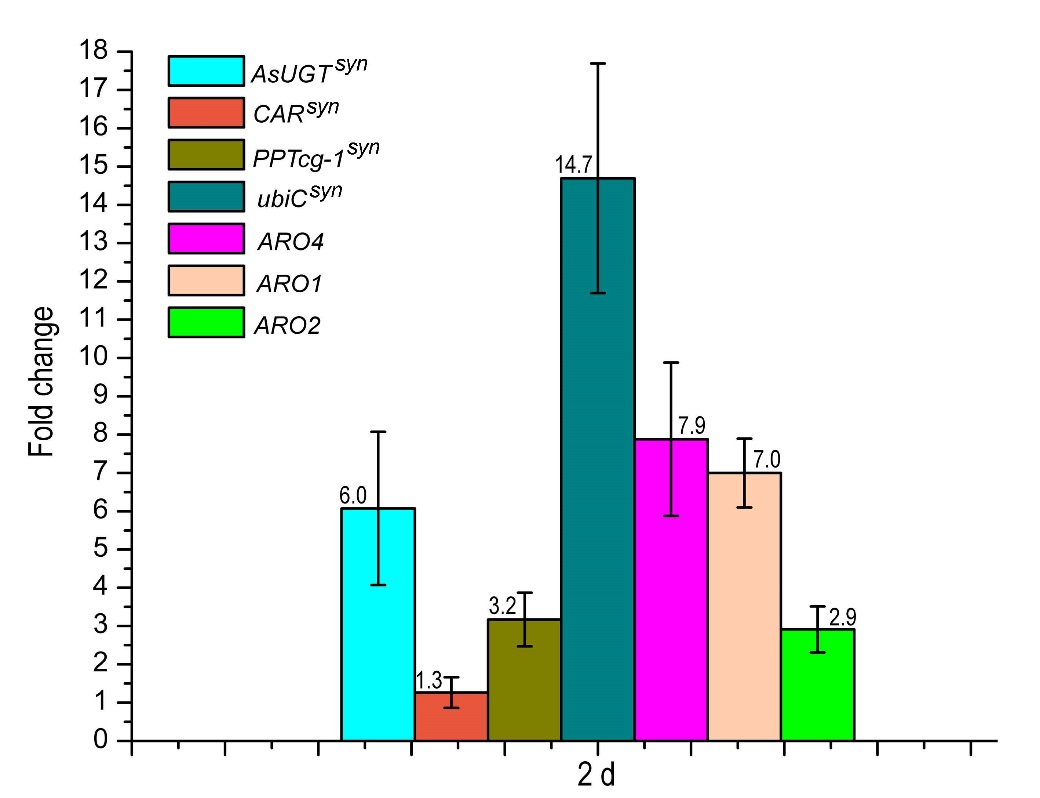


**Figure S5**. The relative transcript levels of target genes in rGS3-PHB compared to rGS3 at 2 day. The fold changes were calculated by the relative quantitative 2^−ΔΔCt^ method and actin was used as the internal standard. The error bars represented standard deviation.

**Table S1** The primers used in this study.

| **Primers** | **Sequence (5′→ 3′)** | **Sources** |
| --- | --- | --- |
| r D-F | CTCCAAAGAGTATCAC | This study |
| r D-R | TGATAAACTCGAACTGGAATTCCAACTTTTTCTTTGGGCATTCGAG | This study |
| r U-F | TGCGCTCGGTCGTTCGGCTCTGCATTCCCAAACAACTCGAC | This study |
| r U-R | AACTTGAAATTGCTGGCCTTTTC | This study |
| r 5.8-F | AATGCCCAAAGAAAAAGTTGGAATTCCAGTTCGAGTTTATC | This study |
| r 5.8-R | AGGTTAATGTCATGATAATAATGGTTTCTTAGGATTCATTAATGCAGCTGGA | This study |
| r 7.4-F | CTAAGAAACCATTATTATC | This study |
| r 7.4-R | TCGAGTTGTTTGGGAATGCAGAGCCGAACGACCGAGCGCAG | This study |
| *δ* U-F | TGTTGGAATAAAAATCAAC | This study |
| *δ* U-R | TAAGGAAAGAGTGAGGAACTAGATACTGGTGAATTTTGAG | This study |
| *δ* D-F | AGAATCAGGGGATAACGCATAAAGAGAATGTGGATTTTG | This study |
| *δ* D-R | TATCATCTACTAACTAGTATTTAC | This study |
| *δ* 7.7-F | TCTCAAAATTCACCAGTATCTAGTTCCTCACTCTTTCCTTAC | This study |
| *δ* 7.7-R | AGAGGTTTTCACCGTCATCAAGTCAGTGAGCGAGGAAGCGGAAG | This study |
| *δ* 6.8-F | FTCCGCTTCCTCGCTCACTGACTTGATGACGGTGAAAACCTCTG | This study |
| *δ* 6.8-R | AGATATTATAACATCTGCAAGTATGCTGTGCTTGGGTGTTTTG | This study |
| *δ* 6.2-F | ACACCCAAGCACAGCATACTTGCAGATGTTATAATATCTG | This study |
| *δ* 6.2-R | TCAAAATCCACATTCTCTTTATGCGTTATCCCCTGATTCTG | This study |

**Table S2** The synthesized genes, sequence of rDNA and *δ* DNA fragment applied in this study.

| **Gene** | **DNA Sequence** |
| --- | --- |
| *AsUGT^syn^* | ATGGAACATACCCCACATATTGCAATGGTTCCAACCCCAGGTATGGGTCATTTAATTCCATTAGTTGAATTTGCAAAACGTTTAGTGTTACGTCATAATTTTGGCGTGACCTTTATTATTCCAACCGATGGCCCATTACCAAAAGCACAGAAAAGTTTTTTAGATGCATTACCAGCAGGCGTGAATTATGTTTTATTACCACCAGTTAGTTTTGATGATTTACCAGCAGATGTGCGTATTGAAACCCGTATTTGTTTAACCATTACCCGTAGTTTACCATTTGTGCGTGATGCAGTTAAAACCTTATTAGCAACCACCAAATTAGCAGCATTAGTGGTTGATTTATTTGGCACCGATGCATTTGATGTTGCAATTGAGTTTAAAGTGAGTCCATATATTTTCTATCCAACCACCGCAATGTGTTTAAGTTTATTTTTCCATTTACCAAAATTAGATCAGATGGTTAGTTGTGAATATCGTGATGTTCCAGAACCATTACAGATTCCAGGTTGTATTCCAATTCATGGTAAAGATTTTTTAGACCCAGCACAGGATCGTAAAAATGATGCATATAAATGTTTATTACATCAGGCAAAACGTTATCGTTTAGCAGAAGGTATTATGGTTAATACCTTTAATGACTTAGAACCAGGTCCATTAAAAGCATTACAGGAAGAAGATCAGGGCAAACCACCAGTGTATCCAATTGGCCCATTAATTCGTGCAGATAGTAGTAGTAAAGTTGATGATTGTGAATGTTTAAAATGGTTAGATGATCAGCCACGTGGCAGTGTTTTATTTATTAGTTTTGGTAGTGGCGGCGCAGTTAGTCATAATCAGTTTATTGAATTAGCATTAGGCTTAGAAATGAGTGAACAGCGTTTTTTATGGGTGGTGCGTAGTCCAAATGATAAAATTGCAAATGCAACCTATTTCAGTATTCAGAATCAGAATGATGCATTAGCATATTTACCAGAAGGCTTTTTAGAACGTACCAAAGGTCGTTGTTTATTAGTTCCAAGTTGGGCACCACAGACCGAAATTTTAAGTCATGGCAGTACCGGCGGCTTTTTAACCCATTGTGGCTGGAATAGTATTTTAGAAAGTGTGGTTAATGGCGTGCCATTAATTGCATGGCCATTATATGCAGAACAGAAAATGAATGCAGTGATGTTAACCGAAGGCTTAAAAGTTGCATTACGTCCAAAAGCAGGTGAAAATGGCTTAATTGGCCGTGTTGAAATTGCAAATGCAGTTAAAGGTTTAATGGAAGGTGAAGAAGGCAAAAAATTTCGTAGTACCATGAAAGATTTAAAAGATGCAGCAAGTCGTGCATTAAGTGATGATGGTAGTAGTACCAAAGCATTAGCAGAATTAGCATGTAAATGGGAAAATAAGATTAGTAGTACCTAA |
| *CAR^syn^* | ATGGCTGTTGATTCTCCAGATGAAAGATTGCAAAGAAGAATTGCTCAATTGTTTGCTGAAGATGAACAAGTTAAAGCTGCTAGACCATTGGAAGCTGTTTCTGCTGCTGTTTCTGCACCAGGTATGAGATTGGCTCAAATTGCTGCTACAGTTATGGCTGGTTATGCTGATAGACCAGCTGCTGGTCAAAGAGCTTTTGAATTGAATACTGATGATGCTACTGGTAGAACATCTTTGAGATTGTTGCCAAGATTTGAAACTATTACATACAGAGAACTGTGGCAAAGAGTTGGTGAAGTTGCTGCTGCTTGGCATCATGATCCAGAAAATCCATTAAGAGCTGGTGACTTTGTTGCTTTATTGGGTTTTACTTCTATCGATTATGCTACATTAGACTTAGCTGATATTCATTTGGGTGCTGTTACTGTTCCATTACAAGCTAGTGCTGCTGTTTCACAATTGATTGCTATTTTGACAGAAACATCTCCAAGATTGTTGGCTTCTACTCCAGAACATTTGGATGCTGCTGTTGAATGTTTATTGGCTGGTACAACACCAGAAAGATTGGTTGTTTTTGATTATCATCCAGAAGATGATGATCAAAGAGCTGCTTTTGAATCTGCTAGAAGAAGATTGGCTGATGCTGGTTCTTTAGTTATTGTTGAAACATTGGATGCTGTTAGAGCTAGAGGTAGAGATTTGCCAGCTGCTCCATTGTTTGTTCCAGATACTGATGATGATCCATTAGCTTTGTTAATATATACCTCTGGTTCTACTGGTACACCAAAAGGTGCTATGTATACAAATAGATTGGCTGCTACTATGTGGCAAGGTAATTCTATGTTACAAGGTAATTCACAAAGGGTTGGTATTAATTTGAACTATATGCCAATGTCTCACATTGCTGGTAGAATTTCTTTGTTTGGTGTTTTAGCTAGAGGTGGTACAGCTTATTTTGCTGCTAAATCTGATATGTCTACATTGTTTGAAGACATTGGTTTAGTTAGACCAACTGAAATTTTCTTTGTCCCAAGAGTTTGTGATATGGTTTTTCAAAGATACCAATCTGAATTGGATAGAAGATCAGTTGCTGGTGCTGATTTGGATACTTTGGATAGAGAAGTTAAAGCTGATTTGAGACAAAATTACTTGGGTGGTAGATTTTTGGTTGCTGTTGTTGGTTCTGCTCCATTAGCTGCTGAAATGAAAACTTTTATGGAATCTGTTCTGGATTTGCCATTACATGATGGTTATGGTTCTACTGAAGCTGGTGCTTCTGTTTTATTAGATAATCAAATCCAGAGGCCACCAGTTTTGGATTATAAATTAGTTGATGTCCCAGAATTGGGTTATTTTAGAACTGATAGACCACATCCAAGAGGTGAATTATTGTTGAAGGCTGAAACAACTATTCCAGGTTATTATAAAAGGCCAGAAGTTACTGCTGAAATTTTTGATGAAGATGGTTTTTACAAGACCGGTGACATTGTTGCTGAATTAGAACATGATAGATTGGTTTATGTCGATAGAAGAAATAACGTTTTGAAGTTGTCTCAAGGTGAATTTGTTACAGTTGCTCATTTGGAAGCTGTCTTTGCTTCTTCTCCATTGATTAGACAAATTTTTATCTACGGTTCCTCTGAAAGATCATATTTGTTGGCTGTTATTGTTCCAACAGATGATGCTTTAAGAGGTAGAGACACAGCTACATTGAAATCTGCTTTAGCTGAATCTATTCAAAGAATTGCTAAGGATGCTAATTTGCAACCATACGAAATTCCAAGAGATTTTCTAATTGAGACAGAACCATTCACTATTGCTAATGGTTTGTTATCTGGTATTGCTAAATTGTTAAGGCCAAATTTGAAGGAAAGATATGGTGCTCAATTAGAACAAATGTATACAGATTTGGCTACTGGTCAAGCTGATGAATTGTTGGCTTTAAGAAGAGAAGCTGCTGATTTGCCAGTTTTAGAAACTGTTTCTAGGGCTGCTAAAGCTATGTTGGGTGTTGCTTCTGCTGATATGAGACCAGATGCTCATTTTACAGATTTGGGTGGTGACTCTTTGTCTGCTTTATCTTTTTCTAATCTGTTGCATGAAATCTTCGGTGTTGAAGTTCCAGTTGGTGTTGTTGTTTCTCCAGCTAATGAATTAAGAGATTTGGCTAATTACATCGAAGCTGAAAGAAATTCTGGTGCTAAAAGACCAACTTTTACATCTGTTCATGGTGGTGGTTCTGAAATTAGAGCTGCTGATCTAACTTTGGATAAATTCATTGATGCTAGAACATTGGCTGCTGCTGATTCTATTCCACATGCTCCAGTTCCAGCTCAAACAGTTTTATTAACAGGTGCTAATGGTTATTTGGGTAGATTTTTATGTCTGGAATGGTTAGAAAGATTGGATAAAACTGGTGGTACATTAATTTGTGTTGTTAGAGGTTCTGATGCTGCTGCTGCTAGAAAAAGATTAGATTCTGCTTTTGATTCCGGTGACCCAGGTTTGTTAGAACATTATCAACAATTGGCTGCTAGAACTTTAGAAGTTTTAGCTGGTGACATTGGTGACCCAAATTTGGGTTTAGATGATGCTACATGGCAAAGATTGGCTGAAACTGTTGATTTGATTGTTCATCCAGCTGCTTTAGTTAATCATGTTTTGCCATATACTCAGTTGTTTGGTCCAAATGTTGTTGGTACTGCTGAAATAGTTAGATTGGCTATTACTGCTAGAAGAAAACCAGTTACATATTTGTCTACAGTTGGTGTTGCTGATCAAGTTGATCCAGCTGAATATCAAGAAGATTCTGATGTTAGAGAAATGTCTGCTGTTAGAGTTGTTAGAGAATCTTATGCTAATGGTTACGGTAATTCTAAATGGGCTGGTGAAGTTTTATTGAGAGAAGCTCATGATTTGTGTGGTTTGCCAGTTGCTGTTTTTAGATCAGATATGATTTTGGCTCATTCTAGGTATGCTGGTCAATTGAATGTTCAAGATGTTTTTACAAGGTTGATCTTGTCTTTGGTTGCTACTGGTATTGCTCCATATTCTTTTTATAGAACAGACGCTGATGGTAATAGACAAAGAGCTCATTATGATGGTTTGCCAGCTGATTTTACTGCTGCTGCTATTACAGCTTTGGGTATTCAAGCTACAGAAGGTTTTAGAACTTATGATGTTTTGAACCCATACGATGATGGTATTTCTTTGGATGAATTTGTTGATTGGTTGGTTGAATCTGGTCATCCAATTCAAAGAATAACAGATTATTCCGATTGGTTTCATAGATTTGAAACAGCTATTAGAGCTTTGCCAGAAAAACAAAGACAAGCTAGTGTTTTGCCATTGTTAGATGCTTATAGAAATCCATGTCCAGCTGTTAGAGGTGCTATTTTGCCAGCTAAAGAATTTCAAGCTGCTGTTCAAACTGCTAAAATTGGTCCAGAACAAGATATTCCACATTTGTCTGCTCCATTGATTGATAAATATGTTTCTGATCTGGAGTTGTTGCAATTATTATAA |
| *PPTcg-1^syn^* | ATGTTGGATGAATCTTTATTTCCTAATTCTGCTAAATTTTCTTTTATTAAAACTGGAGACGCAGTTAATTTGGATCATTTTCATCAATTACATCCTTTGGAAAAAGCATTAGTTGCTCATTCAGTTGATATTAGGAAAGCAGAATTTGGCGACGCAAGGTGGTGTGCACATCAAGCATTACAGGCTTTGGGTAGAGATTCAGGTGACCCAATTTTGAGGGGTGAAAGGGGTATGCCATTGTGGCCATCTTCTGTTTCAGGTTCTTTAACACATACAGATGGTTTTAGGGCCGCGGTTGTTGCTCCTAGATTGTTAGTTAGGTCTATGGGTTTGGATGCTGAACCAGCAGAACCATTGCCTAAAGATGTTTTAGGTTCTATTGCTAGAGTTGGTGAAATTCCTCAATTAAAAAGATTGGAAGAACAAGGTGTTCATTGTGCTGATAGATTATTGTTTTGTGCTAAAGAAGCTACTTATAAGGCATGGTTTCCATTGACACATAGGTGGTTGGGTTTTGAACAAGCTGAAATTGATTTGAGAGATGATGGTACTTTTGTTTCATACCTCTTAGTTAGACCAACTCCAGTTCCTTTTATTTCAGGTAAATGGGTTTTAAGGGATGGTTATGTTATTGCAGCTACAGCTGTTACTTAA |
| *ubiC^syn^* | ATGTCTCATCCAGCTTTGACTCAATTAAGAGCTTTGAGATATTTCAAGGAAATCCCAGCTTTGGACCCTCAATTGTTAGATTGGTTGTTGTTGGAAGATTCTATGACTAAAAGATTCGAACAACAAGGTAAAACAGTTTCTGTTACAATGATTAGAGAGGGTTTTGTTGAACAAAATGAAATTCCAGAAGAGTTACCATTGTTGCCAAAAGAATCTAGGTATTGGTTGAGAGAAATTTTATTGTGTGCTGATGGTGAACCTTGGTTGGCTGGTAGAACTGTTGTTCCAGTTTCTACTTTATCTGGTCCAGAATTAGCTTTGCAAAAATTGGGTAAAACACCATTGGGTAGATATTTGTTTACATCTTCTACTTTGACCAGAGATTTTATTGAAATCGGTAGAGATGCTGGTTTGTGGGGTAGAAGATCAAGATTAAGATTATCTGGTAAACCATTGTTGTTGACAGAATTGTTTTTGCCAGCTTCTCCATTGTATTAA |
| rDNA-up fragment (476 bp) | CTGCATTCCCAAACAACTCGACTCTTCGAAGGCACTTTACAAAGAACCGCACTCCTCGCCACACGGGATTCTCACCCTCTATGACGTCCTGTTCCAAGGAACATAGACAAGGAACGGCCCCAAAGTTGCCCTCTCCAAATTACAACTCGGGCACCGAAGGTACCAGATTTCAAATTTGAGCTTTTGCCGCTTCACTCGCCGTTACTAAGGCAATCCCGGTTGGTTTCTTTTCCTCCGCTTATTGATATGCTTAAGTTCAGCGGGTACTCCTACCTGATTTGAGGTCAAACTTTAAGAACATTGTTCGCCTAGACGCTCTCTTCTTATCGATAACGTTCCAATACGCTCAGTATAAAAAAAGATTAGCCGCAGTTGGTAAAACCTAAAACGACCGTACTTGCATTATACCTCAAGCACGCAGAGAAACCTCTCTTTGGAAAAAAAACATCCAATGAAAAGGCCAGCAATTTCAAGTT |
| rDNA-down fragment (326 bp) | CTCCAAAGAGTATCACTCACTACCAAACAGAATGTTTGAGAAGGAAATGACGCTCAAACAGGCATGCCCCCTGGAATACCAAGGGGCGCAATGTGCGTTCAAAGATTCGATGATTCACGGAATTCTGCAATTCACATTACGTATCGCATTTCGCTGCGTTCTTCATCGATGCGAGAACCAAGAGATCCGTTGTTGAAAGTTTTTAATATTTTAAAATTTCCAGTTACGAAAATTCTTGTTTTTGACAAAAATTTAATGAATAGATAAAATTGTTTGTGTTTGTTACCTCTGGGCCCCGATTGCTCGAATGCCCAAAGAAAAAGTTG |
| *δ* DNA-up fragment (329 bp) | TGTTGGAATAAAAATCAACTATCGGCTGGCAACTAATAGGGACACTACCAATATATTATCATATACGGTGTTAGACGATGACATAAGATACGAGGAACTGTCATCGAAGTTAGAGGAAGCTGAAATGCAAGGATTGATAATGTAATAGGATAATGAAACATATAAAACGGAATGAGGAATAATCGTAATATTAGTATATAGAGATAAAGATTCCATTTTGAGGATTCCTATATCCTCGAGGAGAACTTCTAGTATATTCTGTATACCTGATATTATAGCCTTTACCAACAATAGAATCCCACCAATTATCTCAAAATTCACCAGTATCT |
| *δ* DNA-down fragment (316 bp) | TAAAGAGAATGTGGATTTTGATGTAATTGTTGGGATTCCATTGTGATTAAGGCTATAATATTAGGTATGTAGAAAGTACTAGAAGTTCTCCTCCAGGATTTAGGAATCCATAAAAGGGAATCTGCAATTCTACACAATTCTATAAATATTATTATCATCATTTTATATGTTAATATTCATTGATCCTATTACATTATCAATCCTTGCGTTTCAGCTTCCACTAATTTAGATGACTATTTCTCATCATTTGCGTCATCTTCTAACACCGTATATGATAATATACTAGTAACGTAAATACTAGTTAGTAGATGATAGT |

**Table S3** The real-time PCR primers used in this study.

| Genes | Sequence (5’-3’)  Forward primer | Sequence (5’-3’)  Reverse primer | Amplicon/bp |
| --- | --- | --- | --- |
| *ACT1* | ATGCAAACCGCTGCTCAATC | AAGCTTCTGGGGCTCTGAATC | 103 |
| *AsUGT* | CGATGGCCCATTACCAAAAGC | CGGGTTTCAATACGCACATCTG | 123 |
| *CAR* | ATTTGCCAGCTGCTCCATTG | TGCCACATAGTAGCAGCCAATC | 132 |
| *PPTcg-1* | AAGGTGGTGTGCACATCAAG | ACAGAAGATGGCCACAATGG | 99 |
| *ubiC* | TCTGGTCCAGAATTAGCTTTGC | TACCCCACAAACCAGCATCTC | 121 |
| *ARO4* | ACGGACTTAGCGTCGTAGTTG | TGCATGGTGTTGCTGCTATC | 100 |
| *ARO1* | TCGTGATACGGTTATGGTAGCG | GTACTTGGACAACACGAACACC | 101 |
| *ARO2* | AAAACCGCCACTTACGATGG | TTGGATCAAAAGCGCGTCAG | 135 |
| *ppsA* | CGAGAACGGGCTGAAAATCATC | CAGCTGCGTCATATCGTTTGAG | 118 |
| *tktA* | GTCTACCGACGCATTTGACAAG | AGTCAGCAATACCCGCTTCTAC | 101 |

**Table S4** Information of genes used in this study.

| **Gene** | **P*rotein n*ame** | **Function** | |
| --- | --- | --- | --- |
| *AsUGT* | Glucosyltransferase | | convert 4-hydroxybenzyl alcohol to gastrodin |
| *CAR* | Carboxylic acid reductase | | Convert 4-HBA to 4-hydroxybenzyl aldehyde |
| *PPTcg-1* | Phosphopantetheinyl transferase | | *Activate CAR by phosphopantetheinylation* |
| *ubiC* | Chorismate pyruvate-lyase | | Convert chorismate to 4-HBA |
| *tktA* | Transketolase | | Convert G3P and F6P to E4P |
| *ppsA* | Phosphoenolpyruvate synthase | | Convert pyruvate to PEP |
| *ARO4^K229L^* | Feedback-resistant DAHP synthase mutant, | | Relief repression from tyrosine, condense PEP and E4P to form DAHP |
| *ARO1* | Pentafunctional EPSP synthase | | Convert DAHP to EPSP |
| *ARO2* | Chorismate synthase | | Convert EPSP to chorismate |
